# The Use of Tomographs in Brazil’s National Health System: Case Study on the Efficiency of the Public Network in Rio Grande do Norte

**DOI:** 10.2196/83494

**Published:** 2026-06-01

**Authors:** Tiago de Oliveira Barreto, Elinaldo Bernardo de Oliveira Júnior, Israel José dos Santos Felipe, Jordana Crislayne de Lima Paiva, Nicolas Vinicius Rodrigues Veras, Lorena de Macedo Silva, João Maria Macedo da Costa, Érika Santos de Aragão, Thaísa Góis Farias de Moura Santos Lima, Célio da Costa Barros, Rafael Silveira e Silva, Fernando Rocha de Andrade, Pablo Holanda Cardoso, Andréa Santos Pinheiro, Guilherme Medeiros Machado, Claudia Maria Miranda Veloso, Luciana Simões Camara Leão, Karilany Dantas Coutinho, Ricardo Alexsandro de Medeiros Valentim

**Affiliations:** 1 Laboratory of Technological Innovation in Health (LAIS), Federal University of Rio Grande do Norte (UFRN), Av. Nilo Peçanha, 620 - Petrópolis, Natal, 59012-300, Brazil, 55 84987861321; 2 Institute of Public Health (ISC), Federal University of Bahia (UFBA), Salvador, Brazil; 3 Specialized Health Audit Unit, Federal Court of Auditors, Natal, Brazil; 4 Brazilian Institute of Education, Development and Research (IDP), Federal Court of Auditors, Brasília, Brazil; 5 Public Prosecutor's Office in Rio Grande do Norte (PGR), Federal Public Ministry, Natal, Brazil; 6 LyRIDS, ECE-Engineering School, Paris, France; 7 Research Unit in Governance, Competitiveness and Public Polices (GOVCOPP), University of Aveiro, Center for Global Studies (ESTGA), Aveiro, Portugal; 8 Secretariat of Science, Technology and Innovation, Ministry of Health, Brasília, Brazil

**Keywords:** health economics, RegulaRN Ambulatorial, computed tomography, health regulation, public health

## Abstract

**Background:**

The evaluation of health technologies is fundamental to the sustainability of the Brazilian National Health System (SUS), especially in highly complex and costly procedures such as computed tomography (CT).

**Objective:**

This study analyzed the financial efficiency of CT scanner use in the SUS in the state of Rio Grande do Norte (Brazil), considering the distribution of supply, the profile of providers, and the idle capacity of the public network.

**Methods:**

A total of 49,061 CT scan requests registered in the state system “RegulaRN Ambulatorial” between October 2023 and January 2025 were examined. Subsequently, data cleaning and grouping were performed.

**Results:**

The results indicated that 66.8% (24,769/37,089) of requests were from patients with cancer, 61.2% (22,703/37,089) were women, predominantly from municipalities with higher population densities, such as Natal and Mossoró, with an average waiting time of 44.86 (SD 88.43) days. More than 36,000 (99%) requests for the examinations were performed by private and philanthropic hospitals contracted by SUS, while public units with CT scanners accounted for less than 1% of production, evidencing significant underutilization.

**Conclusions:**

The findings suggest that strategically shifting demand to the public health system can increase efficiency, reduce costs and waiting times, and promote greater equity in access, contributing empirical evidence for more rational public policies on the use of diagnostic technologies.

## Introduction

### Health Economics in the Context of Tomographs in Rio Grande do Norte

Efficiency in public health management is a central and recurring theme in discussions about the sustainability of health systems and the quality of services offered to the population. In Brazil, the Brazilian National Health System (SUS) represents a universal and free model, designed to ensure the constitutional right to health. However, this system faces significant challenges due to the scarcity of financial resources and the constant growth in demand for services, circumstances that intensify the need for rational and efficient allocation of available resources to ensure equity in access and the quality of care provided [1-5].

Within the context of health economics, analyzing the costs related to the state apparatus, as well as monitoring the investment scenario, is essential to promoting efficiency in access to public health [6]. Due to Brazil’s continental size (approximately 8,510,000 km²) and significant population (more than 212 million inhabitants), the country invests billions of Brazilian reais in public health, approximately 3.88% of the federal budget, which leads us to question whether the amount is poorly distributed [7]. In the context of Latin American health systems, Brazil faces persistent challenges in financing public health, with a significant portion of the population depending on services provided by the private sector, despite the universal coverage proposed by SUS [7-9].

Promoting health monitoring through digital health systems can help to address the greatest weaknesses in the public health system [10,11]. One of the important topics for monitoring is the performance of consultations and examinations, especially highly complex examinations. In this sense, computed tomography (CT) is a fundamental clinical procedure for the diagnosis of various diseases, constituting an essential step in many care flows [12]. However, the uneven distribution of this equipment and the variation in the degree of utilization among service providers can compromise both access and the efficiency of the system. To this end, health systems increasingly need timely and efficient regulatory systems.

Recent Brazilian studies highlight regional disparities in the supply and the use of CT scanners in the SUS, as well as pointing out challenges related to production capacity and the allocation of technological resources. Research such as that conducted by Santos [13] and dos Santos et al [14] discusses the availability and degree of the use of CT scanners, whereas investigations carried out by Dovales et al [15] and Viacava et al [16] address issues related to the distribution of resources and the quality of services, which reinforce the urgent need to optimize the use of this equipment in the face of budget constraints and constantly increasing demand.

RegulaRN Ambulatorial is the current system responsible for managing outpatient health care services in Rio Grande do Norte. This digital health system is responsible for coordinating and systematizing requests for tests, while organizing the queues for procedures offered by the SUS. The RegulaRN Ambulatory system incorporates an ecosystem of digital health solutions, unlike other health regulation platforms such as SISREG (National Regulation System) and SIGUS (Outpatient Regulation System), which present problems of interoperability and transparency of information. This paper analyzes requests for CT exams, investigating the execution of this type of procedure for the evaluation of the efficiency of public resource use, in addition to identifying possible regional disparities, efficiency, and effectiveness in the procedures and services provided [17-19].

In the field of health economics, efficiency refers to the ability of a health system to maximize the use of available resources to deliver the greatest possible volume of services and health outcomes. In this context, cost-efficiency is related to the capacity to provide services at lower public expenditure, while maintaining adequate access and quality of care. Evaluating these aspects becomes particularly relevant in the case of high-cost diagnostic technologies such as CT, which require significant financial investment and specialized infrastructure [1].

Given this scenario, the study begins with the following question: what does the analysis of CT scanner utilization and associated public expenditure in the SUS in Rio Grande do Norte reveal: efficiency or waste? In this context, efficiency is understood as the use of equipment to maximize access and clinical outcomes with available resources. Waste, on the other hand, occurs when there is underutilization or inadequate allocation of equipment and resources. The objective is to analyze the use of CT scanners in the public health system of Rio Grande do Norte and to assess the effectiveness of the service, the distribution of supply, the profile of providers, and the adequacy of allocated resources. The relevance of the research lies in providing support for improving the efficiency of public health management in the state of Rio Grande do Norte and proposing strategies that can be replicated in other national or international contexts.

### Sociodemographics and Regional Health Mapping of Rio Grande do Norte

Rio Grande do Norte is a state located in the Northeast region of Brazil, characterized by its socioeconomic and demographic diversity. According to data from the Brazilian Institute of Geography and Statistics [20], this state has approximately 3.3 million inhabitants spread across 167 municipalities and occupies a land area of 52,811 km². The capital, Natal, stands out as the most populous municipality, with over 750,000 inhabitants, followed by Mossoró, with around 264,000 inhabitants, and Parnamirim, with approximately 252,000 residents. The urbanization rate in Rio Grande do Norte is high, reaching 77.8%, while the average population density is 62.54 (DP 34) inhabitants per square kilometer, concentrated in coastal regions.

**Figure 1.**
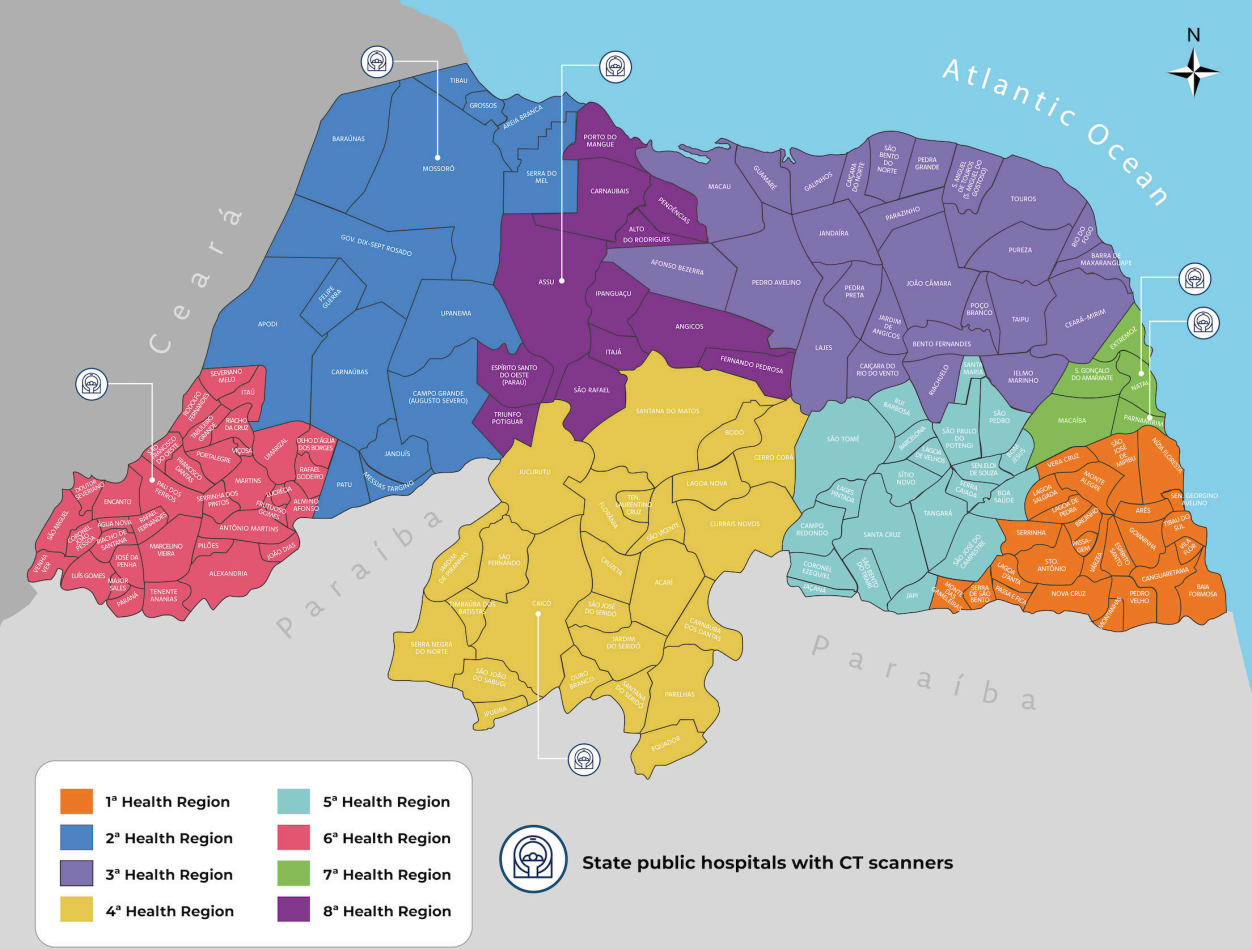
Distribution of Health Regions in Rio Grande do Norte. CT: computed tomography.

In the context of public health, Rio Grande do Norte is currently divided into 8 Health Regions, named in an ordinary manner. Each region has a municipality with greater representation, responsible for managing the health regulation process in the surrounding municipalities. In the first Health Region, the hub municipality is São José de Mipibu, followed in order by Mossoró, João Câmara, Caicó, Santa Cruz, Pau dos Ferros, Natal, and finally, representing the eighth, Assu [21,22]. Figure 1 shows the distribution of Health Regions.

Furthermore, according to data from the National Register of Health Establishments [23] and the government data platform TabNET [24], Rio Grande do Norte has 6173 health units. Of these, 1326 are basic health units under municipal management, 10 are emergency care units, and 21 are state hospitals. Of the hospitals in the state network, 8 are concentrated in the seventh region, 5 in the second region, 2 in the first and fourth regions, and 1 in the third, fifth, sixth, and eighth regions. This configuration reflects the centralization of hospital services in the seventh (Natal) and second (Mossoró) regions, which may influence access to and the provision of specialized services to the population [21,22].

## Methods

### Characterization of the Research Method

This study is characterized as applied research, with a quantitative, descriptive, and exploratory approach, focused on analyzing the use of CT scanners in the state of Rio Grande do Norte. The investigation identifies the distribution of care between public sector health units and contracted private units, considering the potential absorption of demands currently met in both segments, with a focus on rationalization and identification of possible bottlenecks.

The data analyzed were extracted from the RegulaRN Ambulatorial platform, a state system for regulating SUS procedures in Rio Grande do Norte. The database includes requests for CT scans registered between October 2023 and January 2025, with a total of 49,061 records. Each record initially contained 16 variables, which included information about the request, service, provider, and administrative characteristics of the procedure. It should be noted that the platform was structured to migrate data from the platform previously used to regulate state exams. However, these data were not considered in this study because they were not coherent with the study’s objectives.

The RegulaRN Ambulatory system records requests for specialized procedures submitted by health care professionals within the public health network. Each record corresponds to a request for a diagnostic procedure and is associated with an individual patient. However, the same patient may generate multiple requests over time depending on clinical needs, and each request corresponds to a specific examination or procedure. For this study, all CT scan requests registered in the system during the analyzed period were extracted and organized for analysis.

The methodological process was structured in four sequential stages: (1) extraction, in which the original columns from the database were obtained, namely request date, priority, status, patient name, gender, requesting unit, unit municipality, procedure name, subgroup, APAC (Outpatient Health Procedure Authorization) number, authorization date, regulating physician, provider unit, provider municipality, date of care, and date of completion. Following this, we carried out stage (2), feature suitability, in which we preprocessed the temporal variables (dates), which initially had a unified date and time value. Therefore, we separated the date and time and then created derived variables representing the time intervals between the following steps: request and service, request and authorization, authorization and service, and service and closure. In addition, some characteristics were removed, namely columns that contained sensitive variables or that would not contribute significantly to this study, such as the patient’s name, procedure subgroup, APAC number, and name of the regulating physician.

**Figure 2.**
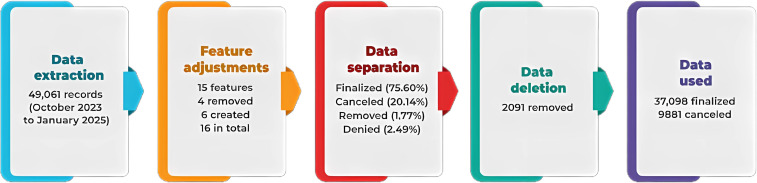
Pipeline created for curating computed tomography exam data from the RegulaRN Ambulatorial system.

It was considered a separation stage (3), given that the status of the requests could contain 4 different types of outcomes, namely completed (37,089/49,061, 75.60% registers), canceled (9881/49,061, 20.14% registers), removed (868/49,061, 1.77% registers), and denied (1222/49,061, 2.49% registers). Thus, this analysis focused on records with completed and canceled status, as they presented greater volume and relevance to the study objectives. Records with removed and denied status were disregarded because they were not significant. Finally, an exclusion stage (4) was included, in which data from outcome variables with removed and denied status were removed, as they did not contribute to the evaluation and objectives of this study. In this regard, the data used for this study consisted of 37,089 completed requests and 9881 canceled requests. Figure 2 shows the sequence of this methodological process.

In order to enable the reproducibility of the methodological process, the anonymized data used to compose this research, within the specified period, can be accessed via the link on Zenodo [25], developed by CERN (European Organization for Nuclear Research), openly and free of charge.

### Ethical Considerations

The requirement for ethical approval was waived by the Research Ethics Committee of the Federal University of Rio Grande do Norte for the studies involving humans because according to Resolution 674/2022 of the National Health Council of the Ministry of Health [26], this research is exempt from registration with the Research Ethics Committee/Brazil or the National Research Ethics Commission (CONEP)/Brazil because, according to the regulations, aggregated and anonymized data do not require an ethics committee review. The studies were conducted in accordance with the local legislation and institutional requirements.

## Results

The results of this survey were organized to consider completed requests and canceled requests separately, in order to provide greater clarity and detail in the analyses presented.

### Requests Completed

When considering the data profile of RegulaRN Ambulatorial, from October 2023 to January 2025, the 37,089 records of exam requests with the status “Completed” show a greater occurrence of data from patients with cancer (in terms of priority), females (in terms of gender), requested mainly by 2 municipalities in the state of Rio Grande do Norte, Natal and Mossoró, for CT of the chest, upper abdomen, and skull (in terms of procedure). Table 1 clearly shows the distribution of request data with the status “Completed.”

**Table 1.** Distribution of data from completed requests (N=37,089).

Feature	Values, n (%)
Categorical values	
Priority	
Oncology	24,769 (66.8)
Hospitalized	2221 (6)
Very high	2654 (7.2)
High	3831 (10.3)
Medium	2999 (8.1)
Low	601 (1.6)
Lawsuit	14 (<0.1)
Sex	
Male	14,386 (38.8)
Female	22,703 (61.2)
Requesting unit	
Health unit 174	8336 (22.5)
Health unit 23	2966 (8)
Health unit 4	1622 (4.4)
Health unit 20	1585 (4.3)
Health unit 19	1535 (4.1)
Others	21,045 (56.7)
Requesting municipality	
Natal	10,815 (29.2)
Mossoró	3691 (10)
Caicó	1133 (3.1)
Others	21,450 (57.8)
Procedure	
Chest CT^a^ scan	9815 (26.5)
Upper abdominal CT scan	9688 (26.1)
Skull CT scan	9158 (24.7)
Others	8428 (22.7)
Service provider unit	
Health unit 4	20,258 (54.6)
Health unit 3	6388 (17.2)
Health unit 12	5264 (14.2)
Health unit 11	1608 (4.3)
Health unit 2	1466 (4)
Others	2105 (5.7)
Municipality provider	
Natal	27,527 (74.2)
Caicó	1621(4.4)
Numerical values	
Request to service	
<30 days	25,256 (68.1)
>30 days	11,833 (31.9)
Request to authorization	
<30 days	27,476 (74.1)
>30 days	9613 (25.9)
Authorization to service	
<30 days	35,716 (96.3)
>30 days	1373 (3.7)
Service to closure	
<30 days	31,512 (85)
>30 days	5577 (15)

a CT: computed tomography.

Regarding time indicators, the average interval between the request and the service was 44.86 days, with a standard deviation of 88.43 days and a median of 20 days (IQR 8-40). The period between the request and authorization had an average of 38 days (SD 86.88 days) and a median of 12 days (IQR 2-32). The interval between authorization and treatment was, on average, 7 days (SD 15.51 days), with a median of 2 days (IQR 1-8). Finally, the average time between admission and discharge was 22.15 days, with a standard deviation of 30.91 days and a median of 14 days (IQR 8-22). These results indicate that the longest delays occur during the authorization and service periods, suggesting that demand exceeds the health care network’s capacity to respond promptly.

Another relevant aspect concerns the distribution of agreements for the provision of examinations. The metropolitan areas of Natal, Mossoró, and Caicó are hubs of reference for other municipalities. Because they have more robust infrastructure, these hub municipalities respond, through agreements, to requests from patients in other municipalities, whether or not those municipalities are located in their Health Region. Thus, Table 2 shows the number of completed requests from municipalities that performed some type of CT scan under agreement with the reference centers.

In addition, it was possible to identify significant patient transfers between regions that either have or do not have health care facilities equipped with CT scanners. Table 3 presents data on the 3 provider units that receive the most requests and the municipalities and Health Regions from which the requests originate. It was possible to identify that health unit 4, belonging to the seventh Health Region of Rio Grande do Norte, expressly serves not only residents of its region but also those of other surrounding regions.

**Table 2.** Distribution of completed requests to service provider units.

Municipality provider	Requesting municipality, n (%)
Natal (n=27,527)	
Natal	10,810 (39.3)
São Gonçalo do Amarante	1102 (4)
Macaíba	1056 (3.8)
Parnamirim	967 (3.5)
Ceará-Mirim	664 (2.4)
Caicó	614 (2.2)
Others	12,314 (44.7)
Mossoró (n=7941)	
Mossoró	3650 (46)
Açu	570 (7.2)
Apodi	367 (4.6)
Baraúna	304 (3.8)
Caraúbas	232 (2.9)
Areia Branca	173 (2.2)
Others	2645 (33.3)
Caicó (n=1621)	
Caicó	519 (32)
Parelhas	163 (10.1)
Currais Novos	134 (8.3)
Apodi	108 (6.7)
Jucurutu	77 (4.8)
Serra Negra do Norte	66 (4)
Others	554 (34.2)

**Table 3.** Distribution of the 3 service providers that receive the most requests and the municipalities/regions from which they are requested.

Service provider unit (Health Region)	Municipality	Municipal Health Region
Health unit 4 (7th)		
Natal	7304	7th
Parnamirim	922	7th
Macaíba	875	7th
São Gonçalo do Amarante	817	7th
Caicó	600	4th
Ceará-Mirim	534	3rd
Santa-Cruz	437	5th
Touros	308	3rd
São José de Mipibu	286	1st
Nova Cruz	275	1st
Health unit 3 (2nd)		
Mossoró	3301	2nd
Apodi	209	2nd
Açu	245	8th
Baraúna	222	2nd
Caraúbas	204	2nd
Areia Branca	121	2nd
Patu	113	2nd
São Miguel	89	6th
Carnaubais	82	8th
Grossos	82	2nd
Health unit 12 (7th)		
Natal	2830	7th
São Gonçalo do Amarante	216	7th
Ceará-Mirim	99	3rd
Macau	95	3rd
Nova Cruz	89	1st
Goianinha	68	1st
Touros	62	3rd
Canguaretama	62	1st
Guamaré	59	3rd
Pedro Avelino	58	3rd

The procedures performed by health care providers are also relevant elements for analysis, especially those identified as the most frequently performed in the respective health care facilities. According to Table 1, health unit 4, health unit 3, and health unit 12 are among the institutions that receive the most patients. It is worth noting that the first 2 units mentioned are references for the care of patients with cancer, representing approximately 66.8% of completed requests. Thus, in a preliminary assessment, it was already expected that these units would have a higher number of requests. In addition, as shown in Table 1, there are 3 tests that are in high demand compared to the others, namely CT scan of the upper abdomen and CT scan of the pelvis, lower abdomen, and chest. Therefore, it is expected that these procedures will also be widely demanded among these units (see Table 4 in Multimedia Appendix 1).

Still, when analyzing which units perform the most different types of procedures, health unit 4 was responsible for performing the most upper abdomen CT scans (5362 in 2024), thoracic spine with or without contrast (81 in 2024), pelvis/lower abdomen (4899 in 2024), face/sinuses/temporomandibular joints (361 in 2014), chest (5790 in 2024), neck (833 in 2024), sella turcica (22 in 2024), and positron emission tomography (PET)-CT (367 in 2024). Health unit 12 performed the highest number of CT scans of the upper limb (26 in 2024) and lower limb (74 in 2024), lumbosacral spine with or without contrast (314 in 2024), and skull (1183 in 2024). Finally, health unit 3 performed the most CT scans of the cervical spine with and without contrast (88 in 2024). A highlight of this analysis is that, in theory, any provider could perform any of the tomography exams, except for PET-CT, which requires a higher level of technology. However, the data show a high distribution in terms of the number of inmates across different units. In addition, not all procedures refer to all units.

We consider analyzing the relationship between the priority of care, the provider unit, and the municipality of the unit that requests the most (Table 5 in Multimedia Appendix 1). Our argument is that it is necessary to identify whether there is any relation or direction between a specific service profile for a specific unit and whether the number of requests for that unit is coming from a nearby municipality or not.

The study found that health units 3 and 4, although they are oncology centers, also address other priorities that could be allocated to other units, which contribute to a better distribution of demand. On the other hand, public hospitals, such as health unit 5 (13 requests) and health unit 9 (87 requests), showed low service levels, with few records, most of them from outside municipalities, suggesting potential for a greater use of this equipment and a reduction in waiting times.

Another important element to highlight is the assessment of which CT scans are most frequently requested over time, as well as their cost, given that the most requested ones do not necessarily incur higher costs for the state government. This will enable the health care network to gain a general understanding of which specific tests are most in demand and, as the network expands, what adjustments need to be made.

According to the data obtained from the RegulaRN Ambulatorial database for 2023 (as of October), CT of the upper abdomen, CT of the pelvis and upper abdomen, and CT of the thorax cost approximately R$ 19,130.94, R$ 18,853.68, and R$ 19,779.45, respectively (Table 4; a currency exchange rate of R$ 1=US $0.199 is applicable). For the year 2024, the most costly and in-demand procedures were also CT of the upper abdomen, CT of the pelvis and upper abdomen, and CT of the thorax, with the significant emergence of PET-CT, costing approximately R$ 1,239,629.46, R$ 1,172,809.80, R$ 1,233,282.81, and R$ 840,780.78, respectively. In January 2025, the same exams remained the most in demand, with costs of R$ 84,287.04 (upper abdomen CT scan), R$ 77,910.06 (pelvis and lower abdomen CT scan), R$ 85,801.89 (chest CT scan), and R$ 52,680.50 (PET-CT scan).

**Table 4.** Representation of the total cost of procedures per year.

Tomography procedure	October 2023 (R$^a^)	October 2024 (R$)	January 2025 (R$)
CT^b^ scan of upper abdomen (R$ 138.63/exam)	19,044	1233.996	83,904
CT scan of lower limb joints (R$ 86.75/exam)	86.75	17,003	2689.25
CT scan of upper limb joints (R$ 86.75/exam)	86.75	5465.25	347
CT scan of the cervical spine with or without contrast (R$ 86.76/exam)	433.8	26,114.76	694.08
CT scan of the lumbosacral spine with or without contrast (R$ 101.10/exam)	1415.4	70,871.1	2628.6
CT scan of the thoracic spine with or without contrast (R$ 86.76/exam)	347.04	21,776.76	1041.12
CT scan of the face/sinuses/temporomandibular joints (R$ 86.75/exam)	1648.25	81,631.75	6072.5
CT scan of the pelvis/lower abdomen (R$ 138.63/exam)	18,853.68	1,172,809.8	77,910.06
CT scan of appendicular segments (arm, forearm, hand, thigh, leg, and foot) (R$ 86.75/exam)	173.5	5638.75	347
CT scan of the sella turcica (R$ 97.44/exam)	0.00	5066.88	292.32
CT scan of the thorax (R$ 136.41/exam)	19,779.45	1,233,282.81	85,801.89
CT scan of the skull (R$ 97.44/exam)	6138.72	368,323.2	16,662.24
CT scan of the neck (R$ 86.75/exam)	1735	96,032.25	7200.25
Positron emission tomography (PET-CT) (R$ 2107.22/exam)	8428.88	840,780.78	52,680.5
Total	78,170.54	5,178,793.09	338,270.56

a A currency exchange rate of R$ 1=US $0.199 is applicable.

b CT: computed tomography.

Regarding the costs associated with performing CT scans, considering that there are public and private health care facilities contracted and paid for with public funds, it is necessary to analyze the distribution of resources used in public health care throughout the period of this study. Finally, analyzing the budget distribution between public and private entities under contract, it was observed that public units (health units 5, 6, 8, and 9) accounted for less than 1% of the total investment in CT scans, with most resources directed to private and philanthropic health care networks (units 1, 2, 3, 4, 7, 10, 11, 12, and 13).

The data show that as of October 2023, the budget for CT scans was R$ 73,170.54 (100%), with the entire amount spent in the private sector. From January to December 2024, R$ 45,366.42 (0.9%) of the cost of the exams was spent in the public sector and R$ 5,133,436.67 (99.1%) in the private sector. Concluding with January 2025, R$ 2,731.74 (0.8%) was spent in the public sector and R$ 335,538.82 (99.2%) in the private sector.

### Canceled Requests

A total of 9881 records of canceled requests were analyzed, the evaluation of which is essential for identifying patterns and possible gaps in the regulatory process. Cancelation may occur at any time, from the request until after service, and is carried out by the requesting unit. Table 8 in Multimedia Appendix 1 details the distribution of cancelations and indicates that the highest concentration occurs in the initial stages of the request, as well as after the service has been provided by the provider unit. This pattern suggests inefficiencies in the early identification of requests that will be canceled, resulting in the unnecessary expenditure of time and human resources in analyzing requests that could have been discarded beforehand.

Following the results, it was also possible to identify that among the canceled requests, there is a greater significance in requests prioritizing patients with cancer and female patients, which was expected given the greater significance throughout the dataset. As for the requesting unit and procedure indicators, there are the following variations: among the canceled requests, the highest number of requests comes from health unit 102, belonging to the municipality of Macaíba, which is located in the metropolitan region of Natal. Although this municipality does not appear significantly in the number of completed requests, it has a higher number of requests canceled in the initial phase. This may represent some difficulty that this municipality faces or has faced in making new requests.

As for the procedure, CT of the upper abdomen, which is one of the most requested examinations, is also among the most canceled. For requests that were only authorized, it should be noted that the requesting units are hospital units, with health unit 32 having the highest recurrence of cancelations after authorization. The most canceled procedure at this stage is chest CT. As for requests that were actually fulfilled, health unit 23, belonging to the municipality of Natal, has the highest number of cancelations. The most canceled procedure at this stage is chest CT.

## Discussion

The discussion of the results obtained in this paper will be conducted from the perspective of the Brazilian Constitution and the outcomes achieved. However, the results obtained indicate the underutilization of public resources in favor of their use in private institutions.

### The Optimization of Health Services as a Constitutional Obligation

The 1988 Constitution of the Federative Republic of Brazil, by establishing the SUS and adopting a model of universal and free access inspired by the British system (Beveridge model), recognized health as a right for all and a duty of the State. Under Article 198 of the Constitution, public health actions and services must form a regionalized and hierarchical network, with decentralized organization and participation by federal entities—the Union, states, Federal District, and municipalities—each of which is responsible for adopting legal, economic, and practical measures aimed at realizing this fundamental right [27].

This constitutional effort to structure the SUS as a federation highlights the joint responsibility of the entities involved in ensuring the provision of quality health services to the population. To this end, public managers are required to perform their duties as efficiently as possible to optimize the relationship between revenue and expenditure. This guideline is reinforced by the principle of efficiency, enshrined in the heading of Article 37 of the Federal Constitution, which should guide all administrative activity.

The existence of essential health equipment and services in a state of idleness is incompatible with the constitutional order and governing legislation, especially given the growing demands of the Brazilian population. This incompatibility becomes even more evident in the case under review when it is noted that, although there is ordinary and continuous demand for CT scans, this demand has been met by the private sector as if it were an exceptional service, while a public network capable of offering such procedures remains idle.

Law number 8,080/1990, which regulates the SUS, establishes in Article 7, items II, IV, and XI that health actions and services must be organized based on the principles of comprehensive care, preservation of personal autonomy, and rational use of resources and that it is the duty of managers to promote the full use of installed capacity, avoid waste, and ensure the efficient use of available resources [28].

The regulation of public health financing, in turn, was consolidated with Complementary Law number 141, of January 13, 2012, which regulates paragraph 3 of Article 198 of the Constitution. The law defined the criteria for apportioning federal, state, and municipal resources, defined what is meant by expenditures on public health actions and services (Article 3), and reaffirmed the minimum revenue requirements set forth in Article 77 of the Brazilian Act of Transitory Constitutional Provisions (ADCT). In addition, Complementary Law 141/2012 reinforced the mandatory use of health funds and emphasized the need for transparent mechanisms for controlling and monitoring public spending (Articles 12, 14, and 16) [29].

Constitutional Amendment Number 86/2015 (known as the “Mandatory Budget Amendment”) reinforced this regulatory framework by establishing a minimum level of federal health spending in the Constitution, making it mandatory to allocate a portion of individual parliamentary amendments to the health sector. The amendment also linked funds from oil royalties to public health actions and services, with the aim of increasing funding and ensuring its stability. In turn, Constitutional Amendment Number 95 of 2016, which was only reversed in 2023, established a freeze on public spending for 20 years, imposing an environment of great fiscal austerity, in which health was heavily impacted by the loss of resources [30].

Given the regulatory landscape of fiscal austerity and the recognized budgetary constraints that limit government action, efficiency in public health management is a constitutional duty, directly linked to the state’s obligation to ensure the fundamental right to health in a universal, equitable, and comprehensive manner. The SUS is facing growing demands and limited resources, so public managers need to make the best use of available resources, getting the most out of public policies and avoiding waste.

In this scenario, the adoption of management practices aimed at rationalizing expenses and enhancing installed capacity becomes imperative. In this case, it was found that, even in the face of continuous and routine demand for CT scans, the State chose to pay for such services from the private network, despite the existence of idle public equipment that was suitable and available to meet the same demand.

Such conduct constitutes flagrant administrative inefficiency and disregard for the constitutional principles of economy, legality, and good governance. Discussing health economics, therefore, is not just a management issue but a legal and ethical requirement, essential for addressing social and regional inequalities. Article 6 of the Federal Constitution establishes health as a fundamental social right, whereas Articles 196 et seq impose on the State the duty to guarantee it based on the principles of universality, equity, and comprehensiveness [2].

Thus, the constitutional order establishes the imperative that public health management acts efficiently, especially in the use of highly complex technologies such as CT, promoting the best use of the existing public infrastructure before resorting, unjustifiably, to the private sector.

Therefore, the search for the optimal path in economic terms, especially with regard to the allocation of highly complex equipment such as CT scanners, is directly linked to the legal duty to promote strategic planning, the evaluation of health technologies, and the maximization of the installed capacity of the public system. The existence of tomography equipment lying idle in public health facilities, when there is demand for the service, not only represents a waste of resources but also constitutes a flagrant affront to the constitutional principles of efficiency, economy, and reasonableness in public administration.

In the specific case of Rio Grande do Norte, the severity of the lack of availability of CT scan services is even more pronounced. As shown in this study, the demand for CT scans is concrete, growing, and well-documented, but it has been met mainly by private providers contracted by the SUS, even in regions where public CT scanners exist. However, the interpretation of underutilization should also consider possible operational constraints, such as equipment maintenance status, workforce availability, and local service organization, which were not directly measured in this study. This finding may indicate operational challenges in the organization of CT services, raising questions about the efficiency of resource allocation and the optimization of existing public infrastructure, which impose on the public administration the duty to optimize resource management—that is, to use the least amount of public resources possible to achieve the highest degree of effectiveness in the service provided.

The allocation of public funds for the contracting of private tomography services, to the detriment of the use of existing public infrastructure, demonstrates a failure to comply with the duty of management optimization, constituting conduct that is uneconomical, inefficient, and inconsistent with the provisions of Article 37 of the Federal Constitution. The allocation of public funds to contract private CT services, while public equipment remains underutilized, suggests potential inefficiencies in resource allocation and highlights the need for improved coordination between available infrastructure and service demand.

### The Inefficiency of Health Care in Rio Grande do Norte: Discussion of the Data Obtained

Considering the scarcity of financial resources within the SUS, efficiency in public management is an essential element for the sustainability of the system. Historically, the public health sector in Brazil has had high health care costs, which can be attributed to multiple factors, such as increased demand, the incorporation of advanced technologies, a shortage of qualified professionals, waste in the health care chain, and institutional management failures. In the case of highly complex exams, such as CT scans, for example, which involve higher public funding costs, monetary management must be more effective [31,32].

The findings of this study reveal that most patients undergoing CT scans through the SUS in the state of Rio Grande do Norte are primarily patients with cancer, with a significant concentration of care in 3 contracted private health care facilities. In view of this, a relevant discussion concerns the possibility of distributing these services to other hospitals, mainly in state hospitals, which, according to the data, have a low number of patients or promote a greater number of consultations for patients without cancer among state hospitals.

It should be noted that, of the 21 state hospitals, 8 of them have CT scan equipment to meet the demand, namely Giselda Trigueiro Hospital, Dr Tarcísio Maia Regional Hospital, Dr Cleodon Carlos de Andrade Hospital, Nelson Inácio dos Santos Regional Hospital, Telecila Freitas Fontes State Hospital, Coronel Pedro Germano Central Hospital, Deoclécio Marques de Lucena Regional Hospital, and Monsenhor Walfredo Gurgel Hospital. However, only 3 of these hospitals performed some type of CT scan to meet the state’s outpatient care demand, and even then, they performed a relatively small number of scans when compared to other units.

According to studies available in academic literature, the minimum capacity of a CT scanner is around 40 exams per day [33,34]. Therefore, the hospitals mentioned above are far below their maximum operational capacity. From this perspective, it is suggested that competent institutions promote a protocol for redistributing the allocation of demands by at least 20% of what is suggested in the literature, not only to reduce public spending but also to reduce average waiting times.

The need to increase the number of appointments at state hospitals in the network is essential to promote greater patient care capacity and minimize waiting times [35]. However, for this increase to be feasible, it is necessary to expand the available human resources, as well as invest in the training and development of the teams, in order to guarantee the quality and efficiency of the service. Given that there is a continuous flow of patients with cancer, those with lower priority in the queue wait considerably longer to be seen to the point that they choose to have the examination conducted at a private facility at their own expense.

From this perspective, the results indicate 2 important points. The first concerns the fact that, although CT scans are considered a high-cost procedure, there are few records of lawsuits, that is, when the patient initiates a procedure with the justice system to make the examination possible. This phenomenon can be explained by the private sector’s ability to offer payment alternatives that facilitate access for patients from the public system. The second aspect concerns the impact of long wait times in public services: patients with a history of public health care services choose to undergo examinations in the private health care network, bearing the costs to ensure faster diagnosis.

Furthermore, from a macroregional perspective, some Health Regions, such as the first, third, and fifth, do not have referral hospitals with CT scan equipment, meaning they do not have the capacity to meet local demand. Therefore, it is necessary for residents to travel a considerable distance so that the nearest providers can serve them. Thus, expanding the capacity of local hospitals to perform these tests could significantly contribute to reducing the need for travel and increasing access.

It was also observed that, among the 13 hospitals that perform CT scans, there is a significant concentration of procedures in a few contracted private units, while the potential idle capacity of the public network appears underutilized according to the available administrative data. This concentration not only leads to increased public costs but also reduces the autonomy of the SUS in providing essential services. As pointed out by Botega et al [36], Brazilian public hospitals often operate below capacity, which reinforces the need for a strategic redistribution of demand to maximize the use of public resources, reduce costs, and foster competition among providers, thereby enhancing the quality and efficiency of care.

As for records of canceled requests, this work presents a considerable volume of requests. In addition, subdividing cancelation periods is also important for identifying where the main bottlenecks lie, given that a request that is made and then canceled for trivial reasons as the days go by systematically contributes to further delays in service. Therefore, institutional intervention is necessary to resolve the main issues presented in the results of this study [37].

Regarding social impact, optimizing the use of CT scanners in public facilities has the potential to expand access to diagnostic exams, reduce waiting times, and minimize waiting times. This effect would be especially relevant for patients classified as lower priorities, who currently experience low service rates in the system. Furthermore, such improvements would not be limited to regions with greater service availability, extending benefits to smaller municipalities and areas lacking dedicated equipment, thus contributing to the promotion of equity in health care access.

The results for Rio Grande do Norte reveal typical challenges faced by public health systems in developing countries, where the concentration of high-cost technologies in a few urban hubs creates territorial inequalities and pressures referral hospitals, resulting in longer waiting times and potential underdiagnosis of diseases. This situation reinforces the importance of strategic planning for the allocation of new equipment, guided by epidemiological needs, population density, and the installed capacity of health units [38,39].

Finally, the results highlight the need to review contracts between the state, municipalities, and service providers, given that some establishments may be serving beyond the necessary demand, while others face difficulties in ensuring adequate access for the population. This review is essential to ensure the equity, efficiency, and sustainability of the public health system in Rio Grande do Norte.

### Conclusions

This study highlights significant distortions in the allocation and utilization of CT scanners in the public health system of Rio Grande do Norte, characterized by the concentration of care in a limited number of contracted private units, despite the existence of underutilized equipment in state public hospitals. These findings suggest the possibility of expanding the installed capacity of the SUS, with potential positive impacts on reducing wait times and optimizing operational costs. Furthermore, it is noteworthy that the future use of analytical tools based on artificial intelligence could provide important support for prioritizing exams and redistributing the health care burden more equitably and efficiently.

The evidence produced in this study can support managers in assessing the absorption capacity of currently outsourced demand, while also reinforcing the importance of more strategic regulation that is sensitive to regional disparities, ensuring equal access to CT scans throughout the state. Regarding limitations, we highlight the incompleteness of data for the entire period between 2023 and 2025, as well as the possibility of inconsistencies in information migrated from previous platforms. The availability of complete historical data would allow for a more comprehensive and contextualized analysis of the performance of service providers. Furthermore, the dataset did not include information on clinical outcomes, diagnostic yield, or patient follow-up, which limits the ability to assess the clinical effectiveness of the performed CT scans. Therefore, to mitigate the impacts arising from the transition between systems in the public sector, we recommend the adoption of periodic audits and data quality frameworks, as recommended by the Department of Informatics of the Unified Health System (DATASUS) [40], to preserve the greatest possible integrity of the information.

For future research, we recommend conducting a longitudinal analysis to assess the effects of restructuring regional contracts, as well as investigating the impact of judicialization resulting from the unavailability of tests. Furthermore, the possibility of replicating the methodology adopted in other high-cost procedures expands the potential contribution to strengthening the rational management of the SUS, promoting greater efficiency and equity in the use of available resources.

## Supplementary material

10.2196/83494 Multimedia Appendix 1 Representation of the number of procedures, distribution of priority care, and distribution of canceled requests.

## Abbreviations

ADCT: Act of Transitory Constitutional Provisions
APAC: Outpatient Health Procedure Authorization
CT: computer tomography
DATASUS: Department of Informatics of the Unified Health System
PET: positron emission tomography
SIGUS: Outpatient Regulation System
SISREG: National Regulation System
SUS: Brazilian National Health System
