# Supplementary material for: The Use of Tomographs in Brazil’s National Health System: Case Study on the Efficiency of the Public Network in Rio Grande do Norte
Source: Online J Public Health Inform. 2026 Jun 1;18:e83494. doi: 10.2196/83494 (PMC13225223; doi:10.2196/83494)
Supplement: Multimedia Appendix 1 [file ojphi-v18-e83494-s001.docx]

**Multimedia Appendix**

Table 4 - Representation of the number of procedures per provider unit

| **Service provider unit** | 2023 | 2024 | 2025 |
| --- | --- | --- | --- |
| Health unit 1 (Private) | 0 | - Upper abdomen: 82 - Lower limb joints: 10 - Upper limb joints: 2 - Cervical spine with and without contrast: 3 - Sacral spine with or without contrast: 20 - Thoracic spine with or without contrast: 2 - Face/cheeks/temporomandibular joints: 18 - Pelvis/lower abdomen: 84 - Sella turcica: 1 - Thorax: 64 - Skull: 100 - Neck: 5 | - Upper abdomen: 10 - Lower limb joints: 2 - Upper limb joints: 1 - Sacral spine with or without contrast: 4 - Thoracic spine with or without contrast: 1 - Face/cheeks/temporomandibular joints: 2 - Pelvis/lower abdomen: 9 - Thorax: 10 - Skull: 20 |
| Health unit 2  (Private) | - Cervical spine with and without contrast: 1 - Sacral spine with or without contrast: 2 - Face/cheeks/temporomandibular joints: 1 - Pelvis/lower abdomen:1 - Skull: 9 | - Upper abdomen: 210 - Lower limb joints: 24 - Upper limb joints: 6 - Cervical spine with and without contrast: 34 - Sacral spine with or without contrast: 110 - Thoracic spine with or without contrast: 24 - Face/cheeks/temporomandibular joints: 83 - Pelvis/lower abdomen: 212 - Appendicular segments (arm, forearm, hand, thigh, leg, foot): 1 - Sella turcica: 3 - Thorax: 236 - Skull: 374 - Neck: 15 | - Upper abdomen: 22 - Lower limb joints: 7 - Upper limb joints: 1 - Cervical spine with and without contrast: 2 - Sacral spine with or without contrast: 13 - Thoracic spine with or without contrast: 3 - Face/cheeks/temporomandibular joints: 15 - Pelvis/lower abdomen: 22 - Thorax: 17 - Skull: 17 - Neck: 1 |
| Health unit 3  (Private) | - Upper abdomen: 13 - Lower limb joints: 1 - Cervical spine with and without contrast:1 - Sacral spine with or without contrast: 2 - Thoracic spine with or without contrast: 1 - Face/cheeks/temporomandibular joints: 3 - Pelvis/lower abdomen: 10 - Appendicular segments (arm, forearm, hand, thigh, leg, foot): 1 - Thorax: 13 - Skull: 2 - Neck: 2 | - Upper abdomen: 1560 - Lower limb joints: 25 - Upper limb joints: 10 - Cervical spine with and without contrast: 88 - Sacral spine with or without contrast: 96 - Thoracic spine with or without contrast:52 - Face/cheeks/temporomandibular joints: 141 - Pelvis/lower abdomen: 1539 - Appendicular segments (arm, forearm, hand, thigh, leg, foot): 11 - Thorax: 1572 - Skull: 745 - Neck: 165 | - Upper abdomen: 98 - Cervical spine with and without contrast: 1 - Sacral spine with or without contrast:1 - Face/cheeks/temporomandibular joints: 8 - Pelvis/lower abdomen: 90 - Thorax: 106 - Skull: 13 - Neck: 13 |
| Health unit 4  (Private) | - Upper abdomen: 106 - Cervical spine with and without contrast: 2 - Sacral spine with or without contrast: 1 - Thoracic spine with or without contrast: 1 - Face/cheeks/temporomandibular joints:9 - Pelvis/lower abdomen:101 - Appendicular segments (arm, forearm, hand, thigh, leg, foot): 1 - Thorax: 115 - Skull: 13 - Neck: 17 - PET-CT: 4 | - Upper abdomen:5362 - Lower limb joints: 19 - Upper limb joints: 12 - Cervical spine with and without contrast: 65 - Sacral spine with or without contrast: 94 - Thoracic spine with or without contrast: 81 - Face/cheeks/temporomandibular joints: 361 - Pelvis/lower abdomen: 4899 - Appendicular segments (arm, forearm, hand, thigh, leg, foot): 18 - Sella turcica: 22 - Thorax: 5790 - Skull: 548 - Neck: 833 - PET-CT: 367 | - Upper abdomen:413 - Lower limb joints: 2 - Cervical spine with and without contrast: 4 - Sacral spine with or without contrast: 6 - Thoracic spine with or without contrast: 5 - Face/cheeks/temporomandibular joints:25 - Pelvis/lower abdomen: 380 - Appendicular segments (arm, forearm, hand, thigh, leg, foot): 3 - Sella turcica: 3 - Thorax: 451 - Skull: 36 - Neck: 65 - PET-CT: 25 |
| Health unit 5  (Public) | 0 | - Upper abdomen:2 - Upper limb joints: 1 - Face/cheeks/temporomandibular joints: 1 - Pelvis/lower abdomen: 2 - Thorax: 4 - Skull: 2 | - Skull: 1 |
| Health unit 6  (Public) | 0 | - Upper abdomen: 47 - Lower limb joints: 4 - Cervical spine with and without contrast: 4 - Sacral spine with or without contrast: 4 - Thoracic spine with or without contrast: 6 - Face/cheeks/temporomandibular joints: 3 - Pelvis/lower abdomen:53 - Appendicular segments (arm, forearm, hand, thigh, leg, foot): 5 - Thorax: 51 - Skull: 36 - Neck: 10 | - Upper abdomen: 1 - Lower limb joints: 2 - Appendicular segments (arm, forearm, hand, thigh, leg, foot): 1 - Thorax: 2 - Skull: 4 |
| Health unit 7 (Private/Philanthropic) |  | - Upper abdomen: 117 - Lower limb joints: 27 - Cervical spine with and without contrast: 7 - Sacral spine with or without contrast: 13 - Thoracic spine with or without contrast: 6 - Face/cheeks/temporomandibular joints: 83 - Pelvis/lower abdomen: 122 - Appendicular segments (arm, forearm, hand, thigh, leg, foot): 4 - Sella turcica:12 - Thorax: 80 - Skull: 525 - Neck: 11 | - Upper abdomen: 7 - Lower limb joints: 2 - Cervical spine with and without contrast: 1 - Thoracic spine with or without contrast: 1 - Face/cheeks/temporomandibular joints: 11 - Pelvis/lower abdomen: 6 - Thorax: 4 - Skull: 41 - Neck: 2 |
| Health unit 8 (Public) |  | - Upper abdomen: 7 - Lower limb joints: 1 - Cervical spine with and without contrast: 1 - Sacral spine with or without contrast: 2 - Thoracic spine with or without contrast: 1 - Face/cheeks/temporomandibular joints: 1 - Pelvis/lower abdomen: 7 - Appendicular segments (arm, forearm, hand, thigh, leg, foot): 4 - Thorax: 13 - Skull: 6 - Neck: 2 | - Upper abdomen: 2 - Face/cheeks/temporomandibular joints: 3 - Pelvis/lower abdomen: 2 - Appendicular segments (arm, forearm, hand, thigh, leg, foot): 1 - Thorax: 1 - Skull: 3 |
| Health unit 9 (Public) |  | - Upper abdomen: 15 - Cervical spine with and without contrast: 1 - Sacral spine with or without contrast: 1 - Thoracic spine with or without contrast: 1 - Pelvis/lower abdomen: 14 - Appendicular segments (arm, forearm, hand, thigh, leg, foot): - Thorax: 40 - Skull: 12 - Neck: 1 | - Upper abdomen: 1 - Pelvis/lower abdomen: 1 |
| Health unit 10 (Private) | - Upper abdomen: 1 - Pelvis/lower abdomen: 1 - Thorax: 1 - Skull: 1 | - Upper abdomen: 386 - Lower limb joints: 9 - Upper limb joints:4 - Cervical spine with and without contrast: 13 - Sacral spine with or without contrast: 34 - Thoracic spine with or without contrast: 11 - Face/cheeks/temporomandibular joints: 37 - Pelvis/lower abdomen: 378 - Appendicular segments (arm, forearm, hand, thigh, leg, foot): 2 - Sella turcica: 4 - Thorax: 412 - Skull: 220 - Neck: 35 | - Upper abdomen: 18 - Upper limb joints: 1 - Sacral spine with or without contrast: 1 - Thoracic spine with or without contrast: 1 - Face/cheeks/temporomandibular joints: 1 - Pelvis/lower abdomen: 15 - Thorax: 19 - Skull: 2 - Neck: 1 |
| Health unit 11 (Private) | 0 | - PET-CT: 32 | 0 |
| Health unit 12 (Private) | - Upper abdomen: 18 - Lower limb joints: 1 - Cervical spine with and without contrast: 1 - Sacral spine with or without contrast: 9 - Thoracic spine with or without contrast: 2 - Face/cheeks/temporomandibular joints: 6 - Pelvis/lower abdomen: 23 - Thorax: 16 - Skull: 38 - Neck: 1 | - Upper abdomen: 1149 - Lower limb joints: 74 - Upper limb joints: 26 - Cervical spine with and without contrast: 83 - Sacral spine with or without contrast: 314 - Thoracic spine with or without contrast: 64 - Face/cheeks/temporomandibular joints: 211 - Pelvis/lower abdomen: 1145 - Appendicular segments (arm, forearm, hand, thigh, leg, foot): 22 - Sella turcica: 5 - Thorax: 708 - Skull: 1183 - Neck: 30 | - Upper abdomen: 36 - Lower limb joints: 1 - Upper limb joints: 1 - Sacral spine with or without contrast: 1 - Thoracic spine with or without contrast: 1 - Face/cheeks/temporomandibular joints: 5 - Pelvis/lower abdomen: 36 - Appendicular segments (arm, forearm, hand, thigh, leg, foot): - Thorax: 19 - Skull: 34 - Neck: 1 |
| Health unit 13 (Private) | 0 | - Upper abdomen: 5 - Lower limb joints: 3 - Cervical spine with and without contrast: 2 - Sacral spine with or without contrast: 14 - Thoracic spine with or without contrast: 3 - Face/cheeks/temporomandibular joints: 2 - Pelvis/lower abdomen: 5 - Thorax: 71 - Skull: 29 | - Lower limb joints: 16 |

Table 5 - Distribution of priority care by provider unit and municipalities with the highest demand.

| **Service provider unit** | **Service priorities** | **Municipalities that request the most** |
| --- | --- | --- |
| Health unit 1 (450) | Oncology: 2 (0.4%)  Hospitalized: 0 (0.0%)  Very High: 143 (31.8%)  High: 213 (47.3%)  Medium: 88 (19.6%)  Low: 4 (0.8%)  Lawsuit: 0 (0%) | Natal: 180  São Gonçalo do Amarante: 39  Macaíba: 20  Nísia Floresta: 16  Nova Cruz: 15 |
| Health unit 2 (1,466) | Oncology: 161 (11%)  Hospitalized: 15 (1%)  Very High: 151 (10.3%)  High: 318 (21.7%)  Medium: 586 (40%)  Low: 235 (16%)  Lawsuit: 0 (0%) | Mossoró: 348  Açu: 239  Baraúna: 82  Pendências: 68  Janduís: 53 |
| Health unit 3 (6,388) | Oncology: 4,507 (70.6%)  Hospitalized: 1,153 (18.1%)  Very High: 93 (1.5%)  High: 298 (4.7%)  Medium: 293 (4.6%)  Low: 44 (0.7%)  Lawsuit: 0 (0%) | Mossoró: 3,301  Apodi: 329  Açu: 245  Baraúna: 222  Caraúbas: 204 |
| Health unit 4 (20,258) | Oncology: 19,095 (94.3%)  Hospitalized: 477 (2.4%)  Very High: 409 (2%)  High: 197(1%)  Medium: 73(0.4%)  Low: 7 (0.03%)  Lawsuit: 0 (0%) | Natal: 7,304  Parnamirim: 922  Macaíba: 817  Caicó: 600  Ceará-Mirim: 534 |
| Health unit 5 (13) | Oncology: 0 (0%)  Hospitalized: 13 (100%)  Very High: 0 (0%)  High: 0 (0%)  Medium: 0 (0%)  Low: 0 (0%)  Lawsuit: 0 (0%) | Currais Novos: 11  Santa Cruz: 2 |
| Health unit  6 (232) | Oncology: 0 (0%)  Hospitalized: 218 (94%)  Very High: 7 (3%)  High: 0 (0%)  Medium: 0 (0%)  Low: 0 (0%)  Lawsuit: 7 (3.02) | Natal: 177  Macaíba: 34  Santo Antônio: 6  Extremoz: 4  Parnamirim: 3 |
| Health unit  7 (1,084) | Oncology: 52 (4.8%)  Hospitalized: 0 (0%)  Very High: 396 (36.5%)  High: 353 (32.6%)  Medium: 243 (22.4%)  Low: 40 (3.7%)  Lawsuit: 0 (0%) | Natal: 225  Macaíba: 59  São Paulo do Potengi: 35  Tangará: 30  São Gonçalo do Amarante: 29 |
| Health unit 8  (57) | Oncology: 0 (0%)  Hospitalized: 57 (100%)  Very High: 0 (0%)  High: 0 (0%)  Medium: 0 (0%)  Low: 0 (0%)  Lawsuit: 0 (0%) | Natal: 55  Currais Novos: 2 |
| Health unit 9 (87) | Oncology: 0 (0%)  Hospitalized: 87 (100%)  Very High: 0 (0%)  High: 0 (0%)  Medium: 0 (0%)  Low: 0 (0%)  Lawsuit: 0 (0%) | Açu: 86  Mossoró: 1 |
| Health unit 10 (1,608) | Oncology: 838 (52.1%)  Hospitalized: 200 (12.4%)  Very High: 242 (15.2%)  High: 115 (7.2%)  Medium: 180 (11.2%)  Low: 32 (2%)  Lawsuit: 1 (0.1%) | Caicó: 519  Parelhas: 163  Currais Novos: 123  Apodi: 108  Jucurutu: 77 |
| Health unit 11 (32) | Oncology: 32 (100%)  Hospitalized: 0 (0%)  Very High: 0 (0%)  High: 0 (0%)  Medium:0 (0%)  Low: 0 (0%)  Lawsuit: 0 (0%) | Mossoró: 8  Natal: 5  Parnamirim: 3  Currais Novos: 1  Janduís: 1 |
| Health unit 12 (5,264) | Oncology: 82 (1.6%)  Hospitalized: 1 (0.01%)  Very High: 1,156 (22%)  High: 2,288 (43.5%)  Medium: 1,492 (28.3%)  Low: 239 (4.6%)  Lawsuit:  6 (0.1%) | Natal: 2,830  São Gonçalo do Amarante: 216  Ceará-Mirim: 99  Macau: 95  Nova Cruz: 89 |
| Health unit 13 (150) | Oncology: 0 (0%)  Hospitalized: 0 (0%)  Very High: 57 (38%)  High: 49 (32.7%)  Medium: 44 (29.3%)  Low: 0 (0%)  Lawsuit: 0 (0%) | Natal: 34  Santa Cruz: 29  Macaíba: 12  Arês: 7  Goianinha: 6 |

Table 8 - Distribution of canceled requests.

| **Features** | **Values (N=9.881)** |
| --- | --- |
| Only upon request (4,812) | |
| Priority | Oncology: 1,156 (24%) |
|  | Hospitalized: 245 (5.1%) |
|  | Very high: 308 (6.4%) |
|  | High: 527 (11%) |
|  | Medium: 606 (12.6%) |
|  | Low: 152 (3.2%) |
|  | Not defined: 1,818 (37.8%) |
| Sex | Male: 1,953 (40.6%) |
|  | Female: 2,858 (59.4%) |
|  | Not defined: 1 (0.02%) |
| Requesting Unit | Health unit 102: 296 (6.2%) |
|  | Health unit 174: 223 (4.6%) |
|  | Health unit 206: 184 (3.8%) |
|  | Health unit 205: 174 (3.6%) |
|  | Health unit 143: 143 (3%) |
|  | Others: 3,792 (78.8%) |
| Procedure | Computed Tomography scan of the upper abdomen:1,102 (22.9%) |
|  | Computed Tomography scan of Pelvis, lower abdomen: 1,071 (22.3%) |
|  | Computed Tomography scan of  Thorax: 936 (19.5%) |
|  | Computed Tomography scan of Skull: 831 (17.3%) |
|  | Computed Tomography scan of the Lumbosacral Spine with or without Contrast: 248 (5.2%) |
|  | Others: 632 (13.1%) |
| Requests that were only authorized (405) | |
| Priority | Oncology: 170 (42%) |
|  | Hospitalized: 180 (44.4%) |
|  | Very high: 17 (4.2%) |
|  | High: 12 (3%) |
|  | Medium: 18 (4.4%) |
|  | Low: 8 (2%) |
| Sex | Male: 149 (36.8%) |
|  | Female: 253 (62.5%) |
|  | Not defined: 3 (0.7%) |
| Requesting Unit | Health unit 32: 49 (12.1%) |
|  | Health unit 35: 20 (4.9%) |
|  | Health unit 27: 20 (4.9%) |
|  | Health unit 47: 19 (4.7%) |
|  | Health unit 29: 16 (4%) |
|  | Others: 281 (69.4%) |
| Procedure: | Computed Tomography scan of  Thorax: 113 (27.9%) |
|  | Computed Tomography scan of  Upper abdomen: 102 (25.2%) |
|  | Computed Tomography scan of  Pelvis / Lower abdomen: 92 (22.7%) |
|  | Computed Tomography scan of  Skull: 55 (13.6%) |
|  | Computed Tomography scan of  Neck: 9 (2.2%) |
|  | Others: 34 (8.4%) |
| Requests that were actually fulfilled (4,664) | |
| Priority | Oncology: 2,116 (45.4%) |
|  | Hospitalized: 751 (16.1%) |
|  | Very high: 600 (12.9%) |
|  | High: 595 (12.8%) |
|  | Medium: 499 (10.7%) |
|  | Low: 92 (2%) |
|  | Not defined:11 (0.2%) |
| Sex | Male: 2,013 (43.2%) |
|  | Female: 2,648 (56.8%) |
|  | Not defined: 3 (0.1%) |
| Requesting Unit | Health unit 23: 267 (5.7%) |
|  | Health unit 174: 232 (5%) |
|  | Health unit 32: 224 (4.8%) |
|  | Health unit 108: 150 (3.2%) |
|  | Health unit 205: 122 (2.6%) |
|  | Others: 3,669 (78.7%) |
| Procedure | Computed Tomography scan of  Thorax: 1.226 |
|  | Computed Tomography scan of  Upper abdomen: 1.094 |
|  | Computed Tomography scan of  Pelvis / Lower abdomen: 1.088 |
|  | Computed Tomography scan of  Skull: 669 |
|  | Computed Tomography scan of  Neck: 122 |
